# Comparison of the Utilization of Tranexamic Acid and Tourniquet Use in Total Knee Arthroplasty: A Retrospective Case Series

**DOI:** 10.7759/cureus.24842

**Published:** 2022-05-09

**Authors:** Promil Kukreja, Brittany M Johnson, Corey Traylor, Kevin J O'Keefe, Sameer Naranje, Jason McKeown, Christopher A Paul, Brooke Bell

**Affiliations:** 1 Department of Anesthesiology and Perioperative Medicine, University of Alabama at Birmingham, Birmingham, USA; 2 Anesthesiology, University of Alabama at Birmingham School of Medicine, Birmingham, USA; 3 Nuffield Department of Orthopedics, Rheumatology and Musculoskeletal Sciences, University of Oxford, Oxford, GBR; 4 Anesthesiology, University of Florida College of Medicine, Gainesville, USA; 5 Department of Orthopaedic Surgery, University of Alabama at Birmingham, Birmingham, USA

**Keywords:** intraoperative blood loss, txa, tourniquet use, total knee amputation, intravenous tranexamic acid

## Abstract

Introduction: Total knee arthroplasty (TKA) carries a high risk for significant blood loss due to bone cuts and extensive soft tissue involvement in the knee region. The use of tranexamic acid (TXA) or a tourniquet are two methods commonly employed to prevent significant blood loss and avoid the need for blood transfusion. TXA has been shown to reduce both intraoperative and postoperative bleeding as well as the probability that a patient will require a blood transfusion. The purpose of this study is to compare the efficacy of TXA and tourniquet use, both alone and in combination, in reducing blood loss during TKA.

Methods: Data for this retrospective cohort study were obtained by searching records of patients who underwent TKA at a tertiary care center from January 2019 to October 2020. Data from 526 subjects were available. A chart review was conducted to determine if the patient received TXA only, tourniquet only, or both TXA and tourniquet during the TKA procedure. Primary outcomes for this study including procedure length in minutes, estimated blood loss in cubic centimeters, and total infusion pressor (phenylephrine) administered intraoperatively in milligrams were recorded for the study. Data were summarized using means and standard errors. Statistical methods used for analysis include one-way ANOVA, probability plots, the Shapiro-Wilk test for normality, the Kruskal-Wallis test, and Tukey’s test.

Results: Data were available for 526 subjects. 122 subjects received tourniquet only (Tourniquet group), 104 received intravenous (IV) TXA only, 264 received both tourniquet and IV TXA (Tourniquet + TXA), and 36 received neither tourniquet nor TXA (None). The groups did not significantly differ in procedure length (p = 0.140) or infusion pressor total (p > 0.20). The groups did significantly differ in estimated blood loss (p < 0.001). Subjects who did not receive either TXA or tourniquet had significantly more blood loss than the Tourniquet and Tourniquet + TXA groups. Similarly, the TXA group had significantly more blood loss than both the Tourniquet and Tourniquet + TXA groups.

Conclusion: This study supports the conclusion that the use of a tourniquet is superior to the use of TXA in reducing intraoperative blood loss during TKA. All groups that underwent TKA using a tourniquet, either alone or in combination with TXA, exhibited significantly lower levels of blood loss compared to the control (no intervention) group.

## Introduction

Total knee arthroplasty (TKA) is a major orthopedic procedure to resurface a knee damaged by arthritis. TKA procedure carries a high risk for significant blood loss due to bone cuts and extensive soft tissue involvement in the knee region [1,2]. There are several risk factors for increased intraoperative bleeding in primary TKA. These include male gender, increased surgical duration, prolonged tourniquet time, certain medication use, age, and hypothermia [3]. Medical conditions associated with increased risk of blood loss include severe chronic kidney disease, end-stage renal disease, cirrhotic liver disease, active malignancy, bleeding diathesis, thrombocytopenia, and anemia [4]. It is vital to maximize hemostasis during surgical procedures such as TKA to promote patient stability, reduce overall morbidity and mortality due to blood loss, and allow for adequate visualization of the surgical field [5]. Patients who experience significant blood loss during TKA may require blood transfusions, which carry the risk of transfusion reactions and secondary infections and increase the cost of patient care [6]. Even if patients do not require transfusion, significant intraoperative blood loss can lead to an increased length of hospital stay, which is associated with an increased risk of hospital-acquired infections and falls, higher healthcare costs for both the patient and the hospital, and increased strain on hospital resources [7,8]. The most common methods to achieve hemostasis and prevent blood loss during TKA are the administration of intravenous or topical tranexamic acid (TXA) and the use of a tourniquet [1,9].

Tranexamic acid (TXA) is a lysine analog that targets the coagulation cascade by blocking the action of plasmin on fibrin, thereby preventing the breakdown of fibrin and reducing both volume and duration of blood loss [10]. TXA has been shown to reduce both intraoperative and postoperative bleeding as well as the probability that a patient will require blood transfusion [9]. In the field of orthopedics, TXA is frequently utilized in a wide range of procedures, including knee, shoulder, and hip arthroplasty, orthopedic trauma surgery, spine surgery, and joint arthroscopies [5]. When given intravascularly, absolute contraindications for TXA use include known allergy to TXA, intracranial bleeding, history of venous or arterial thromboembolism, and active thromboembolic disease [11]. Adverse effects include headaches, nausea, vomiting, diarrhea, seizure, impaired color vision, pulmonary embolism, and deep vein thrombosis [11]. Some studies suggest that TXA may increase the risk of developing acute myocardial infarction in patients with existing cardiovascular risk factors [12]. Several large-scale clinical trials and reviews published over the last decade have shown TXA to be an efficacious and safe drug in many clinical settings. The Clinical Randomization of an Antifibrinolytic in Significant Hemorrhage-2 (CRASH-2) clinical trial showed that early administration of TXA significantly reduces mortality in bleeding trauma patients [13]. The World Maternal Antifibrinolytic (WOMAN) trial found evidence to support the use and safety of TXA for hemorrhaging or high hemorrhage risk obstetric patients [14]. A cumulative meta-analysis of 10,488 patients that examined the effect of TXA on surgical bleeding found that TXA significantly reduced the probability that a patient would require intraoperative blood transfusion [15]. Promising study results combined with the favorable side effect profile and low cost of TXA have led to a recent increase over the last decade in the utilization of the drug in surgical practice [16].

## Materials and methods

The patient population for this retrospective cohort study was obtained by searching the anesthesia records at a tertiary care center from January 2019 through October 2020. The Institutional Review Board of the University of Alabama at Birmingham issued approval number 300000976 for this study. Patients were selected if they underwent total knee arthroplasty. Data were available for 526 subjects. A chart review was conducted to determine if each patient received intravenous (IV) TXA only, tourniquet only, or both TXA and tourniquet during the TKA procedure; primary outcome data were also recorded during the review. Of these subjects, 122 received tourniquet only (Tourniquet group), 104 received IV TXA only, 264 received both tourniquet and IV TXA (Tourniquet + TXA) and 36 received neither tourniquet nor TXA (None). Primary outcomes included procedure length in minutes, estimated blood loss in cubic centimeters, and total infusion pressor (phenylephrine) administered intraoperatively in milligrams.

Data were summarized using means and standard errors (SE). One-way ANOVA was used to compare the four groups. Normality for continuous outcomes was assessed using probability plots and the Shapiro-Wilk test for normality; for any outcomes where normality could not be reasonably assumed, the Kruskal-Wallis test was used in place of ANOVA. When the ANOVA F-test was statistically significant, pairwise comparisons were performed using Tukey’s test. A p-value < 0.05 was considered statistically significant. SAS version 9.4 (SAS Institute Inc., Cary, NC) was used to conduct all statistical analyses.

## Results

**Figure 1:**
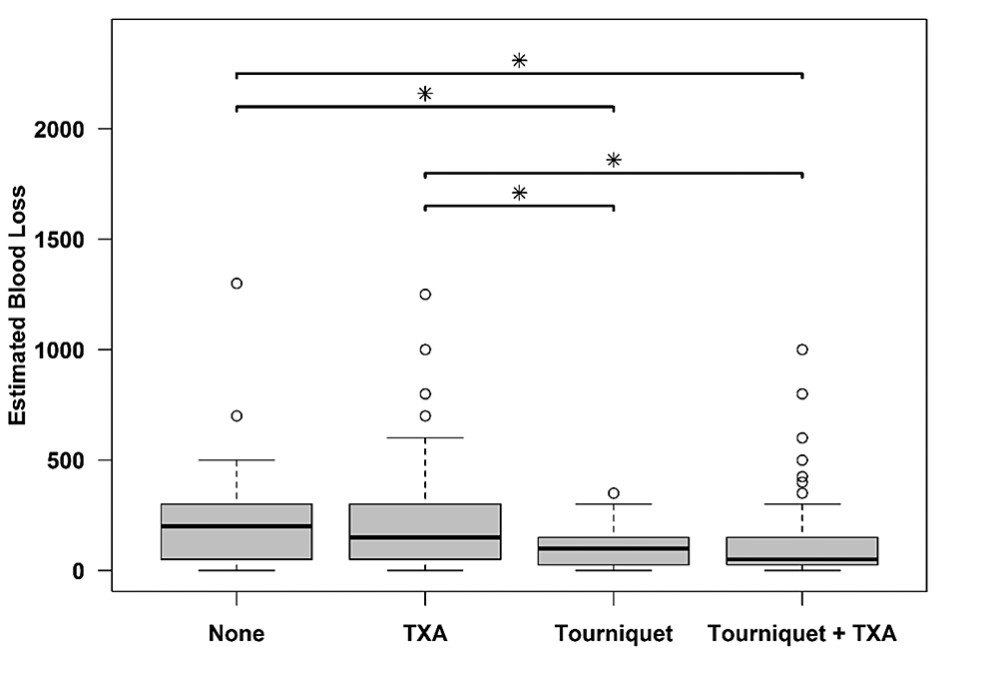
Estimated blood loss in different groups TXA: Tranexamic acid

The groups did not significantly differ in procedure length (p = 0.140) or infusion pressor total (p > 0.20) (Table 1). The groups did significantly differ in estimated blood loss (p < 0.001). Subjects who did not receive either TXA or tourniquet had significantly more blood loss than the Tourniquet and Tourniquet + TXA groups. Similarly, the TXA group had significantly more blood loss than both the Tourniquet and Tourniquet + TXA groups. The Tourniquet and Tourniquet + TXA groups did not significantly differ from each other in estimated blood loss, nor did the TXA and None (no intervention) groups (Figure 1).

**Table 1:** Primary Outcome Data *p-values from one-way ANOVA TXA: Tranexamic acid

Outcome	None (n = 36)	TXA (n = 104)	Tourniquet (n = 122)	Tourniquet + TXA (n = 264)	p*
Procedure length (minutes), mean (SE)	95.17 (3.90)	87.66 (2.25)	91.11 (1.65)	92.82 (1.33)	0.140
Estimated blood loss, mean (SE)	223.42 (40.61)	219.76 (21.25)	104.02 (8.58)	107.51 (8.40)	< 0.001
Infusion pressor total (mg), mean (SE)	0.85 (0.26)	1.05 (0.18)	1.01 (0.16)	4.40 (3.61)	0.831

## Discussion

In this study, the TXA group did not show any benefit over the control (no intervention) group with respect to blood loss. The tourniquet group with and without TXA had significantly less blood loss than TXA or control (no intervention) group.

Prior to the rise in popularity of TXA use, the application of a tourniquet was the primary method used to reduce blood loss during TKA, and it remains popular in current practice [17]. A 2010 survey of orthopedic surgeons found that 95% of surgeons used a tourniquet during TKA [18]. Tourniquets quickly and effectively stop the flow of blood into the surgical field, thus allowing visualization of the surgical field, reducing intraoperative blood loss, and shortening operation times [17]. They are also simple to use, widely available, and relatively inexpensive [19]. Because the use of a tourniquet is a non-pharmacologic intervention, it does not carry any risk of allergic or drug-related adverse reactions as is the case when administering TXA.

The use of TXA in clinical practice may be harmful in patients with a history of coronary artery disease or those having suffered a stroke due to the potential of TXA to promote clotting. The safety concerns are based on the inhibition of fibrinolysis and hence interference of TXA with the coagulation cascade. The potential prothrombotic adverse effects like deep vein thrombosis, myocardial infarction, pulmonary embolism, and ischemic stroke have raised significant concerns for the widespread use of TXA [12]. Porter et al. reported that TXA administration is not associated with an increase in complications in a high-risk patient undergoing knee or hip arthroplasty [20].

However, tourniquet use is not without other risks and adverse effects. The use of a tourniquet leads to significant systemic and local effects as it isolates the extremity from blood circulation [21]. Reperfusion injury of occluded tissue following the removal of the tourniquet is also possible if the tourniquet is applied for upwards of two hours [22]. Strict guidelines for tourniquet use time do not exist, but most recommendations state that patients should be assessed for tourniquet-related complications in surgery after two hours of tourniquet application [23,24]. Nerve injury is the most common complication related to tourniquet use, likely due to both mechanical compression and ischemia; the most common incidences of nerve injury reported are mild and transient, and irreversible nerve damage and paralysis due to extended tourniquet use are possible [25]. Other rare complications related to tourniquet use include compartment syndrome, pressure sores, deep venous thrombosis, consequential pulmonary or venous embolization, and digital necrosis [26]. Tourniquet use is contraindicated in patients with peripheral vascular disease, sickle cell disease, crush injury, diabetic neuropathy, and a history of deep vein thrombosis or pulmonary embolism [23].

Poeran et al. did a retrospective analysis of TXA use and postoperative outcomes in patients undergoing total hip or knee arthroplasty [27]. This study found that TXA had lower rates of allogeneic (compatible donor) or autologous (patient's own blood) transfusion (7.7 % vs 20.1%, p<0.001). TXA group was also found to significantly lower overall complications, need for mechanical ventilation, and admission to the intensive care unit. Overall complications included the event of acute myocardial infarction, among others. If surgical bleeding is reduced by the use of TXA, it may prevent a decrease in hemoglobin and tachycardia, which might avoid insufficient delivery of myocardial oxygen. Our study did not reveal any benefit of TXA with regards to blood loss in total knee arthroplasty patients. We did not report an incidence of post-operative complications like acute myocardial event or stroke.

Many studies and metanalyses comparing the efficacy of TXA alone versus tourniquet alone in primary TKA have been published, as have studies comparing the use of TXA and tourniquet combined versus TXA alone in TKA [28-30]. However, studies comparing the two interventions alone and in combination for a total of four study groups (TXA alone, tourniquet alone, both TXA and tourniquet, no intervention) in the context of TKA are lacking in the current anesthesia literature. This retrospective study gathered patient data from a single tertiary care institute in order to compare the effects of TXA and tourniquet use, both alone and in combination, on the amount of blood loss, length of procedure, and amount of pressor required in primary TKA. We found no difference in length of the procedure (TKA) and amount of pressor used among different groups.

The results of this study contradict the overwhelming benefits of TXA in previous studies in preventing blood loss and related complications. There are many limitations of this study because of its retrospective nature. The adverse effects of TXA and tourniquet were not studied due to a lack of reported data and long-term follow-up. This retrospective study limited our ability to collect data only during the patient’s hospital stay and not beyond hospital discharge for short-term and long-term outcomes related to adverse events and functional recovery. The small number of patients in the control (no intervention) group was also identified as one of the limiting factors. A prospective randomized study is warranted to clearly understand the effect of TXA in preventing blood loss when compared to control (no intervention) or tourniquet use.

## Conclusions

This retrospective study examined the effects of TXA and tourniquet on intraoperative blood loss, length of procedure, and amount of infusion pressor required in the setting of TKA. Between the four study groups (TXA alone, tourniquet alone, both TXA and tourniquet, no intervention), the only significant difference observed was in the effect of TXA and tourniquet on blood loss, with our data supporting the conclusion that use of a tourniquet yielded most beneficial outcomes with respect to intra-operative blood loss. Groups that underwent TKA using a tourniquet or a tourniquet and TXA exhibited significantly lower levels of blood loss compared to the TXA only group and the control (no intervention) group. There was not a significant reduction of blood loss observed when use of TXA alone was compared to the control group. This suggests that there is no added benefit in reducing blood loss by using TXA in the setting of TKA. Our study results contradict the benefits of TXA noted in previous similar studies, and we believe that the findings we report here warrant a future prospective randomized study to fully understand the effect of TXA and tourniquet use in the setting of TKA.
